# Automated calibration of somatosensory stimulation using reinforcement learning

**DOI:** 10.1186/s12984-023-01246-0

**Published:** 2023-09-26

**Authors:** Luigi Borda, Noemi Gozzi, Greta Preatoni, Giacomo Valle, Stanisa Raspopovic

**Affiliations:** Laboratory for Neuroengineering, Department of Health Science and Technology, Institute for Robotics and Intelligent Systems, ETH Zürich, 8092 Zurich, Switzerland

**Keywords:** Reinforcement learning, AI, Automatic calibration, Electrical stimulation, Sensory feedback, TENS, Neurostimulation, Neuropathy

## Abstract

**Background:**

The identification of the electrical stimulation parameters for neuromodulation is a subject-specific and time-consuming procedure that presently mostly relies on the expertise of the user (e.g., clinician, experimenter, bioengineer). Since the parameters of stimulation change over time (due to displacement of electrodes, skin status, etc.), patients undergo recurrent, long calibration sessions, along with visits to the clinics, which are inefficient and expensive. To address this issue, we developed an automatized calibration system based on reinforcement learning (RL) allowing for accurate and efficient identification of the peripheral nerve stimulation parameters for somatosensory neuroprostheses.

**Methods:**

We developed an RL algorithm to automatically select neurostimulation parameters for restoring sensory feedback with transcutaneous electrical nerve stimulation (TENS). First, the algorithm was trained offline on a dataset comprising 49 subjects. Then, the neurostimulation was then integrated with a graphical user interface (GUI) to create an intuitive AI-based mapping platform enabling the user to autonomously perform the sensation characterization procedure. We assessed the algorithm against the performance of both experienced and naïve and of a brute force algorithm (BFA), on 15 nerves from five subjects. Then, we validated the AI-based platform on six neuropathic nerves affected by distal sensory loss.

**Results:**

Our automatized approach demonstrated the ability to find the optimal values of neurostimulation achieving reliable and comfortable elicited sensations. When compared to alternatives, RL outperformed the naïve and BFA, significantly decreasing the time for mapping and the number of delivered stimulation trains, while improving the overall quality. Furthermore, the RL algorithm showed performance comparable to trained experimenters. Finally, we exploited it successfully for eliciting sensory feedback in neuropathic patients.

**Conclusions:**

Our findings demonstrated that the AI-based platform based on a RL algorithm can automatically and efficiently calibrate parameters for somatosensory nerve stimulation. This holds promise to avoid experts’ employment in similar scenarios, thanks to the merging between AI and neurotech. Our RL algorithm has the potential to be used in other neuromodulation fields requiring a mapping process of the stimulation parameters.

*Trial registration*: ClinicalTrial.gov (Identifier: NCT04217005)

**Supplementary Information:**

The online version contains supplementary material available at 10.1186/s12984-023-01246-0.

## Background

Electrical stimulation has been extensively adopted to excite nervous tissue enabling to restore a lost function or to treat disabling pathological conditions affecting the human nervous system. It is widely used in electrophysiological research and clinical applications with very promising results. As a remarkable example, functional electrical stimulation (FES) has been used to induce muscle contractions for restoring functional movements [1], such as gait [2–4] and grasping functions [5, 6]. Another widespread approach, adopted also in clinical practice, is Deep Brain Stimulation (DBS) exploited to relieve the symptoms of Parkinson's disease [7] and to treat epilepsy [8]. More recently, the innovative use of electrical nerve stimulation to artificially restore sensory feedback after limb amputation has shown promising results [9, 10]. The technique exploiting invasive neural interfaces (i.e., implantable electrodes) [11–14] and non-invasive transcutaneous stimulation (i.e., TENS) [15–18] has been successfully tested in upper and lower limb amputees. In addition, TENS preliminary showed good results with pain treatment in people with peripheral neuropathy [19] and affected by reduced peripheral sensitivity with impact on the motor control during movements (e.g., locomotion) [20].

Despite these successful trials, one of the main barriers for clinical adoption of these neurotechnologies is the required calibration (named also mapping or characterization) of the neurostimulation parameters to obtain a desired and effective outcome. The calibration procedure of a sensory neuroprostheses consists of a trial-and-error process, where the neurostimulation parameters are manually changed by a user (e.g., therapist, clinicians, or technicians) according to the produced outcome (e.g., in case of sensory restoration the patient’s answer), with the help of custom-made platforms [21]. This is in contrast with some of other neurotechnologies, as FES, where external (kinematic or EMG) sensors can used in an automated protocol [22, 23]. Indeed, the relationship between the electrical neurostimulation and the desired output is subject-specific, due to the anatomical, perceptual and physiological conditions, requiring a personalized choice of the parameters [22, 24, 25]. The whole process therefore relies mainly on the technical/clinical knowledge and expertise of the experimenter. Furthermore, the neurostimulation parameters may vary over time due to adaptation to stimulation [26] and thus, the patient is forced to go back to the clinic to perform further re-calibration procedures. As a result, patients may undergo inefficient and long recalibrating session as well as unnecessary visits [24, 27]. The multidimensional space of possible parameters (e.g., pulse amplitude, pulse width, train frequency), the changes of the parameters over time, and the need of an expert user, make the characterization a time-consuming, complex and expensive procedure [28].

In the recent past, multiple research groups have tried to automatize the procedure of identifying neurostimulation parameters by exploiting sophisticated algorithms based on artificial intelligence (AI). Feng et al. [29] proposed a closed-loop global optimization technique based on genetic algorithm (GA) to identify novel DBS waveforms that diminish rhythmic, burst-like activity characterizing the Parkinsonian basal ganglia. Lorenz et al. [30] proposed to use non-parametric Bayesian optimization based on relative judgements to search through a large tACS (transcranial alternating current stimulation) parameter space with the aim of identifying frequency-phase combinations that elicit the strongest phosphene perception in subjects. Laferrière et al. [31] showed the use of Gaussian Processes (GPs) based on a hierarchical approach to define optimal inputs for a given EMG target output in the case of multi-electrode stimulation during the motor cortex stimulation.

Nowadays, reinforcement learning (RL) has been successfully applied in multiple fields making its way between the supervised and unsupervised machine learning algorithms. In RL, a software agent makes observations and takes actions within an environment receiving rewards in return. The agent learns, thanks to a positive or negative reward, which are the best actions to undertake in order to achieve a specific goal [32]. Considering neurostimulation, it has been already proposed as the algorithm to identify the optimal stimulation parameters for seizure control in DBS [33] as well as in the optimization of FES parameters for controlling arm movements [23] and cycling [22].

The common for all mentioned uses of AI in neuromodulation is a presence of clearly measurable outcome (e.g., Movement/EMG), therefore in RL context, we can easily assess the state of environment. Instead, if we aim to apply a similar approach in somatosensory prosthetics, technical solutions are to be designed in order to reliably assess the status of it. Indeed, in somatosensory prosthetics, the subject is required to report in detail the electrically-evoked sensation [21, 34, 35]. The resulting quality of the perceived sensation can be captured in a reward that the RL agent can use to optimize the neurostimulation parameters based on the subjects’ feedback in order to evoke a more effective and reliable artificial sensation. RL, acquiring the knowledge by directly interacting with the environment through a trial-and-error process, holds potential for applications that rely on the subject’s answers, such as the sensory feedback restoration using electrical neurostimulation. The goal of this work is to develop a closed-loop system based on RL that allows an automatic, accurate and efficient identification of neurostimulation parameters for sensory neuroprosthetics applications. To this aim, we designed a RL algorithm that selects TENS parameters for sensory restoration based on the induced sensations reported by the subjects. After an offline validation on 49 subjects, the algorithm was integrated with a customized GUI creating an AI-based mapping platform allowing the subject to intuitively interact with the calibrating system. The platform was tested on 15 nerves from five healthy subjects (i.e., peroneal, tibial and sural for each participant), comparing the RL performance with trained and users, as well as with a simplified not AI-based algorithm (i.e., brute force algorithm, BFA). Finally, the RL algorithm was validated in a realistic application on six nerves from two subjects affected by peripheral neuropathy and sensory loss treated through TENS on their lower limbs.

## Methods

### Study design

**Fig. 1:**
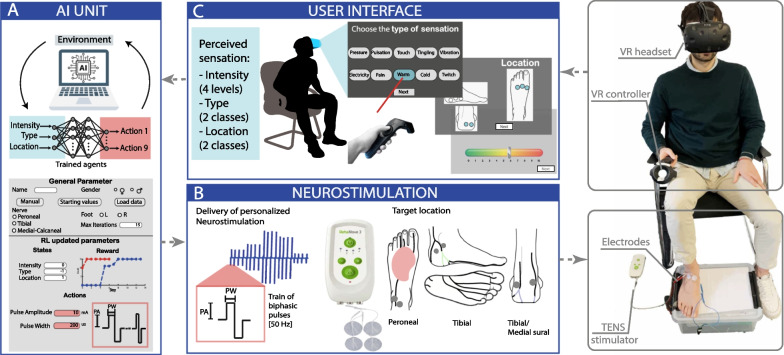
AI-based mapping platform for optimizing the neurostimulation parameters. The subject is interacting with the user interface, simultaneously perceiving neurostimulation selected by the RL algorithm, that is eliciting the electro-touch. The system consists of three parts: the AI brain (**A**), the neurostimulation unit (**B**) and a user interface (**C**). **A** The AI model is an iterative RL machine which initializes and updates the neurostimulation parameters sent to the stimulator. **B** The stimulator receives the parameters and stimulates each of 3 channels accordingly, through a pair of superficial electrodes placed on the skin of the subject in correspondence of the specific nerve. **C** When the stimulation ends, the subject can describe the perceived sensation through comprehensive questionnaires, which include the perceived intensity, type and location of sensation and the intensity of the sensation perceived under the electrodes. The subject’s answers are sent to the AI which: can either finish the characterization, if the desired sensation has been reached or update the neurostimulation parameters and repeat described steps to optimize the sensation

The aim of the study was to evaluate the AI-based mapping platform (Fig. 1) in terms of speed, accuracy, and efficiency in performing the sensory mapping. The RL algorithm was firstly tested offline using a dataset collecting 888 trials from 49 independent subjects with TENS neurostimulation parameters. These data were used to create a data-driven machine learning environment to simulate the perceived sensation of a subject. Offline testing was a key step to test the accuracy and reliability of the RL before moving to online use. Then, the mapping platform was developed for the online testing, integrating the two trained RL agents (Fig. 1A), the neurostimulation system (Fig. 1B) and a user interface (Fig. 1C). In the online implementation, the AI-based mapping platform was tested on 15 nerves of five healthy subjects and later on six nerves of two subjects affected by peripheral neuropathy. Each subject incurred the mapping of three nerves (peroneal, tibial and sural). The purpose of the online testing was to evaluate the mapping performance of the platform by varying contextual factors such as stimulation location and nerve integrity.

### AI-based mapping platform

#### Sensation characterization procedure

The mapping procedure with TENS was performed delivering train of biphasic current pulses with a selected frequency, pulse width and amplitude through superficial electrodes placed on the ankle of the subject in correspondence of the specific nerve. Typically, the pulse amplitude and pulse width values are modulated keeping the frequency value fixed at 50 Hz, based on previous studies [11, 13, 66]. The user selects a reasonable pulse width, and a pulse amplitude ramp is performed until the minimum value that makes the sensation somatotopic is identified. Once the pulse amplitude value is defined, a pulse width ramp is performed to find the minimum and maximum non-painful perceived sensation. In our work, three different nerves of the foot were targeted: peroneal, tibial and sural. Each pair of superficial electrodes were connected to a channel of an electrical stimulator. The device used to release the electric currents was the *RehaMove3* (Hasomed GmBh, Germany), a CE-approved non-invasive four channels surface stimulator. *RehaMove3* has a 0.5mA amplitude resolution in PA and 1us width resolution in pulse width. Therefore, changing first PA provides bigger steps for faster convergence, while the following PW ramp allows a finer and resolute modulation. When the stimulation ended, the subject described the evoked sensation in terms of intensity, type, location and intensity under the electrodes. Two different levels of perceived intensity were identified: just perceivable intensity (or low-level) and strong sensation (or high-level), also defined as the level 2 and level 8 in a scale from 0 (no sensation) to 10 (pain), respectively. The stimulation parameters corresponding to these two levels were chosen in order to have a somatotopic (i.e., perceived distally in the extremity of the foot), natural and minimized in-loco (i.e., under the electrodes) sensation.

#### Closed-loop system

Since the application required to elicit two perceived intensity levels (low and high), two different RL agents have been trained to learn the two individual tasks. The combination of these two agents, named RL algorithm, is therefore the brain of our AI-based mapping platform. The mapping platform was developed for the online testing, integrating the two trained RL agents, the TENS stimulator and user interface. The user interface was developed in *Unity*, a game engine employed to create two or three-dimensional, augmented reality and virtual reality setups. VR, with purposely-designed scenarios and highly-controlled environments, is a widely used tool for neurotechnologies applications [18, 36–39]. Therefore, integrating the stimulation calibration in an AI/VR platform, could be user friendly and intuitive, especially in sight of soon developments of novel light wear and easy to use smart glasses for virtual reality [40, 41]. This platform allowed subjects to directly interact with the algorithm, giving feedback about the evoked sensation (Additional file 2: Movie S1). The VR scenario consisted of an open space environment in which the avatar was seated on a wooden bridge matching the patient's position in the real world. Within this scenario, the patient observed the panels through which he/she could complete the questionnaires describing the sensations evoked by the neurostimulation. The patient’s answer was then collected by the corresponding RL agent and used to optimize the neurostimulation parameters (i.e., stimulation amplitude and pulse width) accordingly. A smart parameter initialization was also integrated into the platform (Fig. 1A). The initial low-level parameters, indeed, were chosen from the dataset (Table 1), based on the subject’s gender and targeted nerve, to ensure higher safety and less discomfort. Thereafter, the following steps were performed:

1. The subject was stimulated by the TENS device (Fig. 1B).
2. The subject described the evoked sensation via the VR environment (Fig. 1C).
3. The patient’s answer was provided to the low-level RL agent which adapted the neurostimulation parameters (Fig. 1A).
4. The previous 1) 2) 3) steps were repeated until the optimal low-level stimulation parameters were obtained.

**Table 1:** Offline dataset overview divided by subject’s gender and targeted nerve

Gender	Number	Nerve	Number
Men	27	Peroneal	552
Women	22	Tibial	108
		Sural	228
Total	49	Total	888

Once the low-level search for the stimulation parameters was completed, the high-level search started. In order to speed up the characterization process, the collected dataset (Table 1) has been exploited for initialization. If the same low-level parameters were found within the dataset (i.e., previous subjects reported similar perceptual thresholds), the corresponding high-level parameters were chosen to initiate the high-level optimization. However, if no match was detected, the low-level parameters were used as the starting point for the high-level search.

### Reinforcement learning (RL)

The AI-based algorithm of the platform is based on the reinforcement learning. This algorithm is formalized through a Markov’s decision-making process (MDP) *(S, A, p, r).* The state transition function p: *S* × *A* × *S → [0, ∞)* gives the distribution of the next state, *S*_*t*+*1*_ based on the current state S_t_ and action *A*_*t*_ [42]. At each time step the agent and the environment interact: the agent receives a representation of the environment’s state, *S*_*t*_* ∈ S*, selects an action *A*_*t*_* ∈ A(s)*, receives a numerical reward *R*_*t*_* ∈ R ⊂* Ɍ, and moves the environment in a new state *S*_*t*+*1*_. The action is chosen following a policy (i.e., a mapping from states to probabilities of selecting each possible action). The goal is to find the optimal policy π* which maximizes the return (i.e., the expected sum of rewards), denoted *G*_*t*_, and defined as:

$${G}_{t}=\sum_{k=0}^{\propto }{\gamma }^{k}{R}_{t+k+1}$$

where γ is a parameter, 0 ≤ γ ≤1, called the discount rate. In our work, we used a Deep Q-Network (DQN) method, a combination of Q-learning, a popular reinforcement learning algorithm, and artificial neural network (ANN), to learn and approximate the optimal state-action function (Q-function). It was firstly proposed by DeepMind to solve a wide range of Atari games [43]. The Q-function estimates the expected cumulative rewards for taking a specific action in a given state. It is a model-free, online, off-policy reinforcement learning method. A DQN agent is a value-based reinforcement learning agent that trains a critic to estimate the return or future rewards [32]. During training, the agent [65] (Additional file 1: Fig. S5):

1. Updates the critic properties at each time step.
2. Explores the action space using ε – greedy policy.
3. Stores past experiences using a circular experience buffer.
4. Updates the critic based on a mini batch of experiences randomly sampled from the buffer (batch updating).

The agent explores the action space using an ε-greedy policy to balance exploration (it chooses random actions with probability ε) and exploitation (selects the action with the highest estimated reward with probability 1 - ε). The agent learns by minimizing the difference between its predicted rewards and the actual rewards it receives. It does this by updating the neural network's parameters using a technique called batch updating. By repeatedly updating the neural network based on its experiences, the DQN agent improves its ability to make better decisions and maximize rewards in the environment.

#### RL implementation

**Fig. 2:**
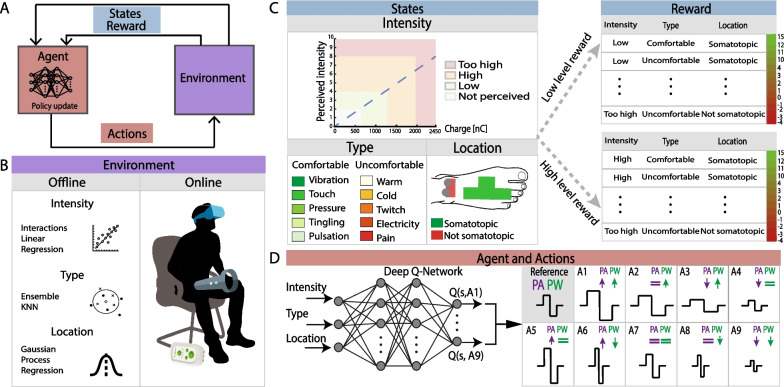
Reinforcement learning (RL) algorithm for sensory neurostimulation optimization. **A** General RL architecture. A software agent observes the environment’s state, take an action moving the environment in a new state and receives a reward in return. **B** During the offline training the environment is simulated through three different machine learning models trained on a dataset of neurostimulation experiments mimicking answers of subjects, for intensity, type and location elicited. In the online condition, the environment is the real subject interacting with the AI-stimulation platform. **C** The states are represented by the intensity, type, and location of the perceived sensation. Each combination of possible states returns a different reward ranging from Min to Max, corresponding to least and most comfortable reported sensations. The definition of the reward function is different for the low-level and high-level agents, responsible respectively to regulate low- and high- levels of reported sensations (Additional file 1: Fig. S3). **D** Each agent is a Deep Q-Network consisting of a neural network with two hidden layers, an input layer with three neurons (states of the environment), and an output layer with 9 neurons (Q-values of possible actions). With a probability of ε, the agent selects a random action (exploration), with probability of 1 − ε, the agent selects the action with the highest Q-value (exploitation). Each action consists of increasing/decreasing/maintaining the same PA and PW of the neurostimulation (nine possible combinations)

The two RL agents (for low and high levels) were trained using the *MATLAB*^*R*^* Reinforcement Learning Toolbox*^*TM*^. For each agent, the key RL elements (Fig. 2A) were defined as follows:

Task: Biphasic and charge-balanced pulse trains at the frequency of 50 Hz (as in previous studies [11, 13, 66]) with a duration of two seconds were used for neurostimulation. The task of the agents was to identify the values of pulse amplitude (PA) and pulse width (PW) to induce a somatotopic and reliable perceivable sensation, while minimizing in-loco sensation. The perceived threshold is indeed proportional to the injected charge, which follows Q = PA * PW. However, as described by the strength-duration curve, it is important to take into account the rheobase (i.e., the threshold current required to excite a neural tissue when the pulse width tends to infinity) and the chronaxie (i.e., the minimum time required for an electric current two times the rheobase to stimulate the neural tissue). However, the rheobase currents and the chronaxie change with the diameter of the sensory fibers [44], distance from the nerve, age [45], and pathological conditions (e.g., significant higher rheobase for polyneuropathy with respect to healthy [46]) among others, making impossible to fix a-priori stimulus current for all tested subjects and pathologies. Therefore, both parameters (PA and PW) affect the stimulation of a neural tissue and have therefore been modulated to create personalized stimulation patterns.

Environment: A simulated environment is a necessary step in developing a RL model. Besides allowing to train the model efficiently without demanding extended training sessions with the patients, simulated environment enables to test different scenarios, conditions, parameters, and models to find the optimal solution. In our real application, the RL environment is the subject himself, that after receiving a new stimulation, changes his/her states (i.e., perceived sensation) accordingly. In the offline training, the environment has to be able to behave as a simulated subject, mapping the relationships between stimulation and perceived sensation. For this purpose, we created a data-driven machine learning environment that learned this relationship from a dataset built from previous neurostimulation experiments carried out on 49 subjects (Table 1). After receiving specific neurostimulation parameters (PW and PA) as inputs, our simulated environment returns as outputs the level of perceived intensity, the type and the location of the elicited sensation similarly to a real subject. Specifically, the environment comprised three different models (Fig. 2B) trained using the MATLAB Classification and Regression Learner toolboxes (See Additional file 1: Sec 1.3): (1) a linear regression interaction model to predict the four levels which describe the perceived intensity (Not perceived/low level/high level/too-high level); (2) an ensemble of a subset of KNN classifiers to predict the two classes in which the type of sensation has been divided (uncomfortable/comfortable); (3) a Gaussian Process Exponential Regression (GPR) to classify the two classes characterizing the sensation location (Somatotopic/Not Somatotopic). Specifically, the GPR builds a probabilistic model for the continuous relationship input-output. Then, to perform binary classification with the GPR, a threshold is chosen to determine the decision boundary; if the predicted probability class exceeds the threshold, the data point is classified as "Somatotopic"; otherwise, it is classified as "Not Somatotopic.". In the online implementation, the environment has been replaced by the recruited subject.

States and reward: States are representations of the environment, and consequently of the patient feedback. Our state is represented by three components which include the information about the perceived intensity, type and location of the electrically-evoked sensation. The combination of their values gives a finite number of possible states theoretically equal to 16. However, the perceived intensity level *not perceived* was considered as a single state because the patient would not be able to describe the type and location of a not-perceived sensation.

Overall, the final number of states was equal to 13 (Fig. 2C). The reward is linked to the state reached following the action chosen by the agent. We tested different reward functions (Additional file 1: Fig S1). Specifically, a discrete nonlinear custom reward showed better performance during training. To define the reward, the states were ordered prioritizing (1) intensity (2) location (3) type of the sensation. Since the target level of perceived intensity was different between low and high level, the states and the corresponding reward have therefore been ordered differently for the two conditions (Additional file 1: Fig. S3A, S3B). Specifically, in order to push the agent to learn actions which result in the best evoked somatotopic sensation and, more importantly, to avoid actions which provoked patient’s discomfort we attributed an increasing negative reward for the uncomfortable states and increasing positive reward for the desired ones. A zero reward was instead assigned to the *not perceived* state. During the offline training, a phase of fine-tuning of the reward function was carried out, proving that the selected one led to the best results (Additional file 1: Fig. S1E).

Agent: The trained agents were two Deep Q-Networks, each of them designed with 3 neurons in the input layer, 9 neurons in the output layer and respectively 40 and 30 neurons in the hidden layers (Fig. 2D). The number of input neurons is equal to the number of elements that characterize a state of the environment (i.e., perceived intensity, type, location). The number of output neurons is instead equal to the number of possible actions that the agent can choose.

Actions: The action is the way through which the agent interacts with the environment. The agent’s purpose is the modulation of PA and PW. Two arrays have been defined: a PA array made of 16 values ranging from 1 to 16 mA; a PW array made of 54 values ranging from 70 µs to 600 µs. The resolution of each array was 1 mA and 10 µs, respectively. The maximum and minimum values were chosen based on those in the previously acquired dataset (Table 1). The agent can either modulate only PA, only PW or both PA and PW simultaneously. Specifically, The PA value could be kept fixed, increased or decreased by 1mA. The PW value could be kept fixed, increased or decreased by 10 µs. (Fig. 2D). The agent learned which action to perform depending on the received reward. The PA and PW are characterized by a different resolution in charge (and therefore perceived intensity). Modulating either one or both simultaneously allows the RL to perform larger or smaller steps towards the optimal state, according to the distance from it.

### Offline implementation: training and testing

The dataset used for offline training comprised data collected from calibration previously performed by expert experimenters (Table 1). The mapping was executed following the procedure previously described (section “AI-based mapping platform, “Sensation characterization procedure*”*) on 27 men and 22 women for a total of 49 subjects. The number of targeted nerves (i.e., peroneal, tibial or sural) varied according to the type of experiment. For each nerve, a different number of stimulations was performed. The total number of previously collected trials available was 888. Each trial was composed by the stimulation parameters (PA, PW) and the respective feedback reported by the subject (intensity, type and location of sensation) together with personal information (weight, gender…). These data were used to build a simulated environment based on data-driven machine learning algorithm, able to simulate the perceived sensation (type, intensity and location) of a specific combination of stimulation parameters. It allowed to train the model efficiently without demanding extended training sessions with each subject.

Each training episode involved the simulation of a characterization carried out on each subject of the offline dataset, to identify the stimulation parameters for the desired intensity level. When an episode ended, another one immediately started simulating a different and randomly initialized subject (e.g., different weight, gender…). This allowed agents to gain insight into the variability between subjects. In the offline training the pipeline worked as follow: (1) the new actions were given as input to the offline environment (composed by the three previously trained ML models to mimic patients perceived sensation, see Methods—RL implementation); (2) The simulated environment returned the respective new states of the system (perceived intensity, location and type); (3) The reward was computed from the new states; (4) the agents optimized new actions. An episode could end in two ways: (1) the agent reached a state where the perceived intensity level was classified as too high which implied the failure of that episode; (2) the maximum number of iterations was reached which implied the success of the characterization. On the other hand, the training could end when the maximum number of episodes was reached, when an average cumulative reward was exceeded, or it could be stopped manually. The hyperparameters characterizing the agents' training were: (1) Learn rate = 0.0001; (2) L2 Regularization Factor = 0.0001; (3) Target smooth factor = 0.001; (4) Discount factor = 0.99; (5) Mini batch size = 64; (6) Epsilon = 1; (7) EpsilonDecay = 0.005. These parameters were the default parameters of DQN agents in *MATLAB*^*R*^* Reinforcement Learning Toolbox*^*TM*^ [65] and they have already been proposed in other studies [47–51]. Specifically, the learn rate and L2 regularization factor were regularizing the updates of the weights during the critic’s learning process, the target smooth factor was controlling the rate at which the target network's (used to generate the target Q-values for training) parameters were updated and the discount factor was scaling down the rewards to keep the total sum of rewards bounded. Epsilon(ε) and EpsilonDecay, instead, have been used to balance the exploration-exploitation mechanisms of the policy, which pushes the agent into exploration phases at the beginning of training while exploiting what he has learned towards the end. After training, the agents were then tested offline before moving on to the online implementation. New simulations were carried out using the trained agents and evaluating their performance based on the final state reached and the number of steps required by the agents to converge. During the testing phase, each episode ended when the parameters chosen by the agent did not change for five consecutive iterations, which meant that the agent considered that state as the maximum achievable. A final state was then considered correct when it reached the target level of perceived intensity (low- or high-level). The testing phase was carried out on the entire dataset in order to evaluate the agents’ performance on each of the simulated subject.

### Online implementation

#### Subjects’ recruitment

Five healthy subjects (3 females, 2 males; 24.4 ± 1.5 years old) were recruited (Table 2). Two neuropathic subjects (male) with consequent distal sensory loss were recruited. For each participant, three different nerves were tested. Two expert users (i.e., researcher used to frequently perform the characterization of neurostimulation parameters in different neuroprosthetic applications) and five naïve experimenters (biomedical engineers that never performed a neurostimulation characterization before) were involved.

**Table 2:** Online healthy and neuropathic dataset used to validate the RL algorithm

Healthy	Number	Nerve	Number	Neuropathic	Number	Nerve	Number
Men	2	Peroneal	5	Men	2	Peroneal	2
Women	3	Tibial	5			Tibial	2
		Sural	5			Sural	2
Total	5	Total	15	Total	2	Total	6

#### Healthy subjects: experimental protocol

Fifteen different nerves from five independent subjects were mapped. Specifically, peroneal, tibial and sural nerves were tested, which are the relevant innervation areas for the use of lower-limb neuroprosthetic devices [11, 13]. Firstly, the experimenter placed the electrodes on the subjects’ ankle to correctly target the three selected nerves. Then, the characterization (low- and high-levels) was performed by: (I) an expert user with technical knowledge and frequent experience in mapping the neurostimulation (simulating the therapist); (II) a naive experimenter who never performed the characterization before (simulating a non-experienced doctor in the clinic); (III) the RL-algorithm; (IV) a brute force algorithm (BFA). The expert and naïve characterization were performed using a designed GUI [34]. Short pulse trains of electrical current varying in PA and PW were selected by the experimenter. The volunteers described the sensation in terms of type, location, extent and intensity. The process ended when the low- and high-level stimulation parameters were found. The expert (i.e., the experimenter already trained in performing manual neurostimulation calibration) performed the characterization of each stimulation area following the standard procedure (Additional file 1: Fig. S6) enriched by the experience gained by previous characterization. Indeed, the expert begins the characterization by performing a pulse amplitude ramp at a fixed PW value but with personalized choice of starting PA and PW step, intentionally selected based on the technical knowledge and experience (Additional file 1: Sec. 3, expert mapping algorithm). Then, the expert adapts the ramp selecting different steps for PA and PW based on the reported sensation by the subject (e.g., if the subject is not perceiving any sensation, the expert will select a larger starting value and step for the next stimulation). Adapting the initial, final and step values of the ramp allow the expert experimenter to perform a reliable but fast characterization. The naïve experimenter followed a clearly defined protocol (Additional file 1: Fig. S6). The BFA was implemented using the VR system and UNITY 3D as coding platform. The BFA algorithm performed monotonic increasing ramps of PA and PW until the optimal parameters were found (Additional file 1: Fig. S7). The parameters were initialized at the lowest charge that was able to elicit a low-level sensation in 49 subjects of the offline dataset, to ensure generalizability and to avoid failure of the algorithm in case of too strong perceived sensations. Indeed, since the BFA cannot decrease the stimulation charge once the target intensity is exceeded, it might fail in case of too high initialization. The parameters found for the low-level were then used as starting values to find the high-level parameters. The RL-algorithm was implemented in the AI-based platform previously described. The AI mapping platform allows the user to directly perform the characterization without any help from the experimenter. The four conditions (i.e., expert user, naïve experimenter, RL-algorithm, BFA) were randomly presented to the subjects. Each subject was tested two times, 1 week apart, to evaluate the performance of the algorithm over time and its ability to overcome the problem of the adaptation to stimulation. The position of the electrodes on day 1 was saved to ensure the repeatability of the experiment during day 2 and to allow the stimulation parameters to be initialized starting from those found on day 1.

#### Diabetic subjects: experimental protocol

The AI-based mapping platform was then tested on six nerves of two individuals suffering from peripheral neuropathy associated with peripheral sensory damage and loss. During this testing phase the characterization of the peroneal, tibial and sural nerves has been performed on each subject on day 1 only.

#### Evaluation metrics

Mapping time: For each nerve mapped, the start and final time of the characterization have been recorded. The recorded time include both the stimulation time (choice of the parameters and delivery of the stimulation) and the time required by the subject to report the perceived sensation in terms of intensity, type and location.

Number of delivered stimulation trains: For the expert and naive conditions, the minimum, maximum and the step chosen for the PA and/or PW ramps were saved during the experiments. The number of delivered stimulations have been calculated afterwards. For what concerns the BFA and the RL algorithm specific counters were updated each time new stimulation parameters were delivered.

Injected charge: The charge value was calculated as the product between the pulse amplitude and pulse width values found (Q = PA * PW).

Sensation Quality index: The quality index is a measure defined to evaluate the quality of the mapping during characterization. After each characterization, the subjects filled a form to describe the evoked sensation in terms of perceived intensity, type, location and intensity perceived under the electrodes. Then, the quality index takes into account if: (i) the desired perceived intensity level (I) has been reached (low or high); (ii) The type of sensation (T) belonged to the comfortable or uncomfortable class; (iii) The location (L) of the sensation was somatotopic and if the in-loco sensation (SE) was higher or lower than the intensity of the somatotopic one. Different weights were attributed to the individual component so that their total sum equals 1 in the best case. Then, we defined each single weight (intensity, location and type) to achieve a perceivable, somatotopic, and comfortable sensation. It is first required that the electrically evoked sensation is clearly perceived and identified, and, for this reason, we prioritize the intensity (w_1_ = 0.6).

Furthermore, the modulation of the intensity in electrical sensory feedback applications has shown promising results (e.g., grasping of an object with a prosthetic hand [16, 52, 53], or walking phase information relating to the pressure exerted by a lower limb prosthesis [13]). Second, it is important to evoke a somatotopic sensation that is inherently simple and intuitive, allowing for immediate and effortless understanding of the feedback [16], hence a medium weight was given to the location (w_3_ = 0.25). Third, the type of sensation was introduced to avoid uncomfortable sensations but, since inducing natural (touch-like) sensory feedback with non-invasive interfaces is still a unresolved challenge [54–56], the lowest weight was assigned to it (w2 = 0.15).

The value of the quality index ranged from 0 to 1, as follows:

$$Q={w}_{1}*I+{w}_{2}*T+{w}_{3}*L*\left(1-\frac{SE}{10}\right)\,\text{with}{:}\, {w}_{1}=0.6, {w}_{2}=0.15, {w}_{3}=0.25$$

### Data collection and statistical analysis

Plotting, data processing and analysis were performed in Matlab (R2020b, The MathWorks, Natick, MA, U.S.A.). Statistical analysis was performed using built-in Matlab functions. Bar plots that present results from statistical analysis show the mean and standard deviation of the mean. For healthy subjects’ results asterisks on plots indicate the following statistical significance levels: p < 0.0083 (*), p < 0.0017 (**), p < 0.00017 (***). The normality of the distributions has been checked using the Kolmogorov-Smirnov test. A nonparametric Friedman’s test to compare the experimental condition on outcome measures was used. Since we were conducting a hypothesis test with multiple comparisons (i.e., four conditions tested for a total of six possible combinations), a post-hoc analysis with Wilcoxon signed-rank tests was conducted with a Bonferroni correction applied, resulting in a significance level set at p < 0.0083. For diabetic subjects’ results a Mann-Whitney test has been used to compare the average value of the outcome measures between healthy and diabetic subjects. Asterisks on plots indicate the following statistical significance levels: p < 0.05 (*), p < 0.01(**), p < 0.001 (***).

## Results

### RL agents achieve high offline accuracy

The RL architecture for sensory neurostimulation optimization and the key elements (environment, states, reward, agent and actions) are shown in Fig. 2. The two RL agents (for low and high levels) were trained using the *MATLAB*^*R*^* Reinforcement Learning Toolbox*^*TM*^. An RL environment to train and validate the algorithm offline was properly created. Indeed, a simulated environment based on data driven machine learning models trained on previously collected data was created, necessary for long and time-consuming training of the RL. The offline environment was fitted using previously collected data (888 trials from 49 subjects, Table 1). More details on the design and training of the simulated environment are reported in Additional file 1: 1.2. Specifically, the three machine learning algorithms (Fig. 2B, Additional file 1: Fig. S1B) that made up the environment (see Methods—RL implementation) were: (1) a linear regression interaction model which reached a RMSE << 0.001 to predict the perceived intensity level (2) a KNN ensemble classifier with an accuracy of 72.3% to predict the type of sensation (3) a Gaussian Process Exponential Regression model used for binary classification which reached a final accuracy of 91.8% to predict the location of sensation. In this way, the simulated environment was able to mimic the subject’s answer in terms of perceived sensation during offline implementation. After the training, the two agents were tested offline evaluating the accuracy of the system and the number of steps to converge. Convergence was considered achieved when the parameters did not change for five consecutive iterations. A state was considered correct if the desired level of perceived intensity was reached. The low-level agent showed an accuracy of 88.51% (Additional file 1: Fig. S1C) and an average number of steps equal to 19.3 ± 15.3 (Additional file 1: Fig. S1D). On the other hand, the high-level agent showed an accuracy of 98.64% (Additional file 1: Fig. S1C) and an average number of steps equal to 9.93 ± 22.4 (Additional file 1: Fig. S1D). Examples of mapping simulations are shown in Additional file 1: Fig. S2.

### AI-based mapping platform performs as a trained experimenter in peripheral nerve stimulation

**Fig. 3:**
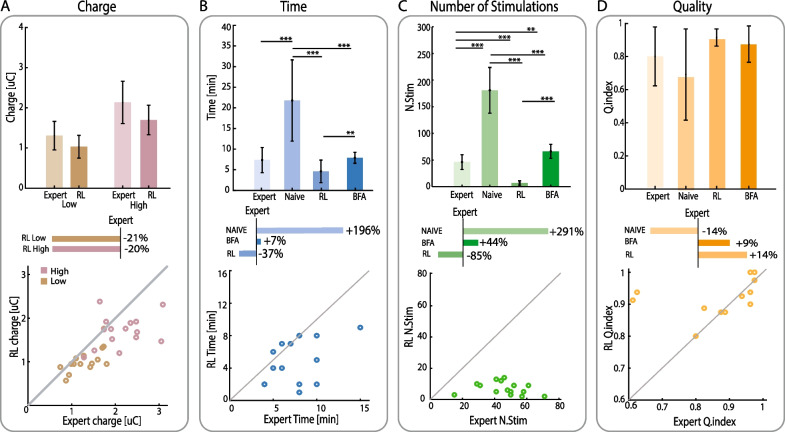
Performance of the RL-based algorithm, BFA algorithm, expert user and naïve user. The results during the first day of characterization are shown. These plots are computed for: **A** final charge released by the stimulation parameters found, divided by low level and high-level calibration, comparing expert and RL performance. The RL was then compared to the expert for both low and high level in terms of percentage of improvements. **B** Time needed to perform the characterization of the nerve, **C** number of stimulations delivered and **D** overall sensation quality of the mapping. The bar plots represent the mean values and standard deviation of the measurements of 15 nerves of five independent subjects for the 4 approaches (p < 0.0083 (*), p < 0.0017 (**), p < 0.00017 (***)). The four conditions were then compared to the expert and expressed as a percentage of the expert performance. The scatter plots represent a direct comparison between the RL and the expert for each trial

Figure 3 shows the results obtained on healthy subjects during day 1 of the experiments. The performance has been evaluated in terms of mapping duration, number of delivered stimulation trains, injected charge, and sensation quality. No statistically significant difference between Expert (i.e., trained experimenter, see methods) and RL-algorithm was found in the amount of charge injected for low and high levels (Fig. 3A). However, considering only the average result in the two conditions, it is possible to notice a trend. The RL presents an average decrease of the injected charge of 21% for the low-level and 20% in the high level. Indeed, considering all the characterizations performed on day 1, in 86% of cases for the low-level and in 80% of cases for the high-level the charge injected by the RL-algorithm was lower than that of the Expert. This trend is also repeated during day 2, although the difference was not statistically significant (Additional file 1: Fig. S4A). Regarding the results of the other evaluation metrics, considering all four experimental conditions, we can assert that the RL algorithm was the fastest to perform the sensation mapping of the 15 nerves compared to the Naive (Wilcoxon, p < 0.00017) and BFA (Wilcoxon, p < 0.0017) conditions requiring similar time compared to the expert (RL: 4.6 ± 2.7 min; Exp: 7.3 ± 3.0 min; Wilcoxon p > 0.0083) (Fig. 3B). Although no statistical significance emerged in the comparison with the Expert, the results showed again a promising trend. Among the three conditions of which performance was evaluated with respect to the Expert (i.e., RL, Naive and BFA) the RL was the only one that on average presented a decrease in time. Indeed, comparing the results obtained in characterizing the 15 nerves, in 74% of cases the RL took less time than the Expert thus proving that the RL on average required less time to characterize the same target nerve (i.e., peroneal, tibial, sural) (Fig. 3B). A similar pattern was observed in the other evaluation metrics. The RL released the lowest number of stimulations (RL: 7.1 ± 4.0 stimuli), with a statistically significant difference compared to the Expert (Wilcoxon p < 0.00017), Naïve (Wilcoxon p < 0.00017) and BFA (Wilcoxon p < 0.00017) conditions (Fig. 3C). The number of stimulations decreased by 85% compared to the Expert (46.2 ± 13.9 stimuli). Furthermore, in all the characterizations performed (i.e., 100% of cases), the RL released a lower number of stimulations than those released by the Expert. Finally, the quality of the mapping performed by the RL was similar to the others experimental conditions (i.e., Expert, Naïve, BFA) (Wilcoxon, > 0.0083) (Fig. 3D). The comparison with the expert again showed how, although not statistically significant (Wilcoxon, p > 0.0083), on average the quality of the mapping achieved by the RL was higher than the expert, reporting in 53% of the characterizations performed a sensation quality index higher than the latter. On the second day of testing, the RL algorithm showed the same trend as on day 1, being again the fastest condition in performing the mapping, requiring the least number of stimulations, and obtaining high sensation quality index (Additional file 1: Fig. S4).

### AI-based mapping platform properly characterizes neuropathic nerves

**Fig. 4:**
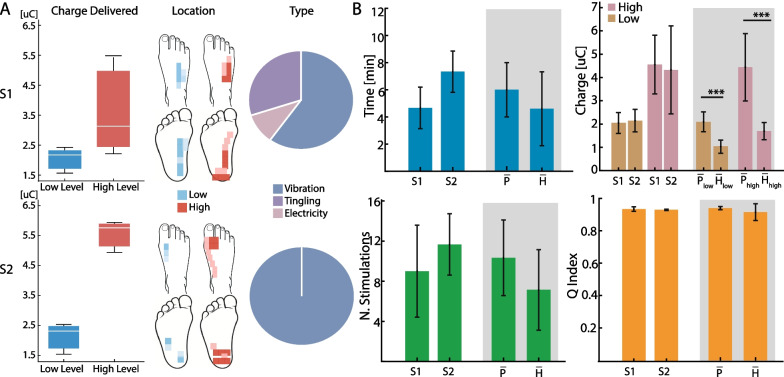
RL-based platform adapts to impaired nerves of peripheral neuropathic subjects. The results obtained by performing the mapping of the three nerves (i.e., peroneal, tibial and sural) on two neuropathic subjects (S1 and S2) using the AI mapping platform are reported. **A** Results of the characterization in terms of injected charge for the low and high level, location and type of evoked sensations. **B** RL performance in terms of average and standard deviation over the three nerves reporting time, number of stimulations delivered, injected charge (low- and high-level calibration) and quality of the mapping for each subject. Bar plots in the shaded area represents the average values of each metric for people affected by polyneuropathy ($\overline{P }$) and healthy participants ($\overline{H }$) for easier comparison (p < 0.05 (*), p < 0.01(**), p < 0.001 (***))

The AI-mapping platform was able to successfully identify parameters for neuropathic subjects. Figure 4A shows the mapping characterization for subject 1 and subject 2 respectively. The results are reported in terms of injected charge values for the low- and high-level, sensation location elicited over the foot and type of evoked sensation. The average time to perform the mapping of a neuropathic nerve was 6 ± 2 min requiring a number of stimulations on average equal to 10.3 ± 3.8 (Fig. 4B). The average quantity of charge injected was equal to 2.1 ± 0.4 µC and 4.4 ± 1.4 µC for low and high level respectively (Fig. 4B) and the quality of the mapping reached a sensation quality index value on average equal to 0.94 ± 0.01 (Fig. 4B). No differences emerged in the comparison between healthy and diabetic subjects in terms of time, number of stimulations released and sensation quality index (Mann-Whitney, p > 0.05). On the other hand, an increase equal to 103% for the low-level and 162% for the high-level in the charge released in diabetic subjects compared to that released in healthy subjects emerged (Mann-Whitney, p < 0.001) (Fig. 4B).

## Discussion

The optimization of neurostimulation parameters is a required step of applications exploiting electrical neurostimulation [22, 24, 25, 27]. Specifically, in the case of sensory feedback restoration with peripheral nerve stimulation (e.g., TENS), the quality and naturalness of the restored sensation are strongly dependent on the choice of the optimal parameters [34, 35]. Indeed, the neurostimulation parameters are subject-specific, they are sensitive to different nerve conditions (e.g., in case of nerve tissue damage in people affected by peripheral neuropathy [57, 58]), and may change over time due to neural adaptation [26] and/or displacement of the electrodes. Usually, the stimulation parameters are manually selected by an experimenter and adapted accordingly to the subjective perceived sensations. This procedure is expensive and time-consuming and mainly relies on the experience of the experimenter. Furthermore, the subject is forced to come back to the clinic every time a further calibration is needed [24, 27, 35].

In this work, we presented an AI-based platform able to perform an automatic mapping of the neurostimulation parameters for sensory feedback restoration achieving performances comparable to a trained experimenter. Compared to other applications where the output was objectively quantifiable [22, 23], defining an objective measure of a reported sensory feedback is an important challenge. Thanks to our platform we were successfully able to quantify the sensory feedback so that it could be used by a properly designed RL algorithm.

We validated the platform both in offline and online scenarios. Firstly, we tested the algorithm offline to evaluate the performance of the low-level and high-level RL agents. Both agents have accurately identified stimulation parameters for TENS in a limited number of steps. Overall, the high-level agent showed better performance than the low-level agent, likely due to the different type of initialization of the stimulation parameters for the two agents. Indeed, while the low-level agent started the optimization from the lowest possible pair of parameters available in the original dataset, the high-level agent started from the parameters previously found by the low-level agent. Thanks to a better personalized initialization, the high-level agent required fewer steps to converge and achieved higher accuracy in finding the target state. Moreover, in order to compensate for different transition probabilities deriving from dissimilar perceptual thresholds among the subjects, training and validation of the two RL-agents was performed on a dataset collected on a total of 49 individuals. Offline testing therefore proved that the two RL-agents were able to find an optimal common policy among the subjects and that RL system was accurate and reliable in the identification of neurostimulation parameters.

Then, the online testing phase on healthy subjects confirmed that the RL algorithm was able to perform an automatic characterization with performance comparable to a trained experimenter. The RL algorithm required comparable time to an expert experimenter, released a comparable quantity of charge but with a substantially lower number of stimulations. These last two factors are important to reduce the long-term tissue damage and the energy consumption in battery-powered wearable devices [59, 60]. The comparison with the naïve condition highlighted the problems arising from no experience in performing the calibration. Indeed, this condition presented the highest number of stimulations and time required. Our platform would therefore allow the subject to perform a reliable and fast calibration in complete autonomy, avoiding returning to the clinic with a consequent impact on time and costs [28]. We also compared the RL algorithm with a Brute Force Algorithm, to evaluate whether a simpler algorithm, not based on AI, was still able to achieve similar results. Despite being both automatic, RL outperformed BFA for all evaluation metrics. Indeed, while the BFA was based on a linear increment of the parameters of the stimulation, the RL algorithm learned the optimal policy from the data, following a learning paradigm based on experience similar to an expert.

Furthermore, to test the inter-day repeatability of the RL calibration, the same healthy subjects were tested a second time after 1 week, with acquired knowledge of the stimulation parameters. The second day of experiments also showed that the RL algorithm was the fastest condition while maintaining a high mapping quality and releasing a comparable quantity of charge with respect to the expert condition. This testifies the repeatability of the RL approach over different environment conditions and the effectiveness in recalibration getting closer to a subject-specific characterization.

Finally, very encouraging results were also obtained on six nerves of two neuropathic individuals with reduced nerve integrity. Although the algorithm was trained on healthy data, it was able to successfully complete the calibration with high sensation quality and maintaining a low number of stimulations delivered and time required. The injected charge was higher for neuropathic than healthy nerves both for high and low levels, due to nerve damage caused by peripheral neuropathy, in agreement with previous works [57, 58].

Interestingly, the restoration of sensory feedback in other body areas (e.g., upper limb sensory feedback [16, 35, 61] or with different neurostimulation technologies (e.g., not invasive [34], intraneural [13, 35, 62, 63], epidural [64] and intracortical [65]) follow the same characterization paradigm proposed in this work. Specifically, the main stimulation parameters are pulse amplitude, pulse width and frequency and the outcome are measured in term of location, intensity and quality. Therefore, our results support the potential of the RL algorithm in other neuroprosthetics applications [66].

Although we have shown that we can perform the mapping in a completely automatic way, this is only true in case of subjects with a known position of the surface electrodes. Finding the placement of the TENS electrodes in order to obtain a somatotopic sensation remains a time-consuming procedure, particularly when sensory deficits are present. To make the subject totally independent from the experimenter, it is essential to make also the electrode placement automatic. A possible solution to the problem could be the use of a matrix of electrodes placed on the foot. Following the choice of the most effective pair of electrodes, the algorithm could be applied to the specific stimulation channel to consequently perform the mapping of the parameters. This would allow to automate both the electrode placement and the optimization of the stimulation parameters. One aspect to consider in this study is the definition of BFA automatic mapping, which has certain limitations. The BFA was defined to replicate the naïve calibration method, employing short PW steps to prevent any discomfort and unpleasant sensations. In future works, it will be necessary to explore different non-AI automatic calibration approaches that could incorporate more intelligent parameters selection. Nevertheless, expanding the space of stimulation parameters, such as introducing the option to choose a pair of electrodes, would exponentially escalate the complexity of BFA and its optimal definition.

Another limitation of the study lies in the number of subjects affected by peripheral neuropathy which has been tested. The number of subjects should drastically increase to evaluate the performance of the RL algorithm considering also different degrees of lesion. Indeed, one of the main future objectives is acquiring further data to improve the parameters initialization phase based on the biometric data of the subject (e.g., degree of lesion, body mass index, etc.). The choice of the initial parameters directly influences the number of stimulations required and consequently the time needed to perform the characterization. Being able to initialize the parameters based on the subject's biometric data would therefore allow the platform to be safer, faster, and more accurate. Furthermore, the calibration process could further benefit from a personalized training and designing of the RL algorithm. Indeed, a personalized RL would allow specific state transition probability for each subject (also depending on the degree of lesion), possibly improving the time and number of steps required to calibrate. However, this would be possible only after multiple days of use of the generalized common RL algorithm, which will collect data specific to the subject that can be used to fine tune and personalize the algorithm. Furthermore, the default hyperparameters were selected for RL training. While this is a common approach during the initial development of a machine learning application, fine-tuning of the RL hyperparameters could potentially improve the performance of our automatic calibration. Given the majority of positive rewards in the actual reward function, the RL agent was driven towards longer training episodes over shorter training episodes. Future steps will therefore also be directed towards further optimization and fine-tuning of the actual reward function, which could eventually improve the time and number of steps of RL calibration. However, the results obtained so far are a good indication for future tests and open the way to the solution of a topical problem such as the optimization of the neurostimulation parameters. In particular, a significant future step towards the use of such technology in daily life will encompass the development of an AI system-on-chip designed to be portable and with a user-friendly interface.

## Conclusion

In this work, we presented the RL algorithm applied for the characterization of sensory feedback with TENS to optimize and automatize the choice of stimulation parameters. The platform showed promising results both on non-pathological and pathological (i.e., neuropathic) nerves. In both cases it was able to successfully perform the mapping automatically, in a fast manner, delivering a low number of stimulation trains and low injected charge, while maintaining a high quality of the mapping, and outperforming other methods. The testing with different subjects and nerves, over different days has proven the RL algorithm generalizability and repeatability in mapping. That makes it a promising tool for the standardization of a subject-specific procedure such as sensation characterization. This represents a step towards a platform that will allow subjects to autonomously optimize the stimulation parameters without the need of an expert. This will diminish in-person visits to the clinic to perform further recalibration phases, thus saving time and costs, while maintaining the same health benefits.

### Supplementary Information

**Additional file 1: Fig S1.** RL environment algorithms and offline testing results. **Fig. S2.** Step-wise evolution of offline simulated RL. **Fig S3.** Low- and high-level agents states and rewards. **Fig S4.** Online comparison of RL, BFA, expert and naïve mapping performances. **Fig S5.** Deep Q-learning training algorithm. **Fig S6.** Naïve mapping algorithm. **Fig S7.** Brute force mapping algorithm.

**Additional file 2: Movie S8.** Explanation of the AI-VR calibration platform for sensory feedback.

## Data Availability

*MATLAB*^*R*^ codes and data showing the interaction with the RL platform and its step-wise evolution are available at the following directory: https://github.com/noemi-gozzi/RL-somatosensory-calibration.git (https://doi.org/10.5281/zenodo.7648810). The datasets generated during and/or analysed during the current study are available from the corresponding author on reasonable request.

## Abbreviations

TENS: Transcutaneous electrical nerve stimulation
GUI: Graphical user interface
BFA: Brute Force Algorithm
RL: Reinforcement learning
AI: Artificial intelligence
FES: Functional electrical stimulation
EMG: Electromyography
VR: Virtual reality
DQN: Deep Q-Network
PA: Pulse amplitude
PW: Pulse width
ML: Machine learning
KNN: K-nearest neighbors
RMSE: Root mean squared error

## References

[1] Rushton DN (2003). Functional electrical stimulation and rehabilitation—an hypothesis. Med Eng Phys.

[2] Belda-Lois J-M (2011). Rehabilitation of gait after stroke: a review towards a top-down approach. J Neuroeng Rehabil.

[3] Robbins SM, Houghton PE, Woodbury MG, Brown JL (2006). The therapeutic effect of functional and transcutaneous electric stimulation on improving gait speed in stroke patients: a meta-analysis. Arch Phys Med Rehabil.

[4] Ferrante S (2016). A personalized multi-channel FES controller based on muscle synergies to support gait rehabilitation after stroke. Front Neurosci.

[5] Thrasher TA, Zivanovic V, McIlroy W, Popovic MR (2008). Rehabilitation of reaching and grasping function in severe hemiplegic patients using functional electrical stimulation therapy. Neurorehabil Neural Repair.

[6] Popovic MR, Popovic DB, Keller T (2002). Neuroprostheses for grasping. Neurol Res.

[7] Perlmutter JS, Mink JW (2006). Deep brain stimulation. Annu Rev Neurosci.

[8] Fisher RS, Velasco AL (2014). Electrical brain stimulation for epilepsy. Nat Rev Neurol.

[9] Raspopovic S, Valle G, Petrini FM (2021). Sensory feedback for limb prostheses in amputees. Nat Mater.

[10] Raspopovic S (2020). Advancing limb neural prostheses. Science.

[11] Petrini FM (2019). Sensory feedback restoration in leg amputees improves walking speed, metabolic cost and phantom pain. Nat Med.

[12] Valle G, Saliji A, Fogle E, Cimolato A, Petrini FM, Raspopovic S (2021). Mechanisms of neuro-robotic prosthesis operation in leg amputees. Sci Adv.

[13] Petrini FM (2019). Enhancing functional abilities and cognitive integration of the lower limb prosthesis. Sci Transl Med.

[14] Preatoni G, Valle G, Petrini FM, Raspopovic S (2021). Lightening the perceived prosthesis weight with neural embodiment promoted by sensory feedback. Curr Biol.

[15] Pan L, Vargas L, Fleming A, Hu X, Zhu Y, (Helen) Huang H (2020). Evoking haptic sensations in the foot through high-density transcutaneous electrical nerve stimulations. J Neural Eng.

[16] D’Anna E (2017). A somatotopic bidirectional hand prosthesis with transcutaneous electrical nerve stimulation based sensory feedback. Sci Rep.

[17] Chai G, Sui X, Li S, He L, Lan N (2015). Characterization of evoked tactile sensation in forearm amputees with transcutaneous electrical nerve stimulation. J Neural Eng.

[18] Risso G, Preatoni G, Valle G, Marazzi M, Bracher NM, Raspopovic S (2022). Multisensory stimulation decreases phantom limb distortions and is optimally integrated. iScience.

[19] Gibson W, Wand BM, O’Connell NE (2017). Transcutaneous electrical nerve stimulation (TENS) for neuropathic pain in adults. Cochrane Database Syst Rev.

[20] Najafi B, Talal TK, Grewal GS, Menzies R, Armstrong DG, Lavery LA (2017). Using plantar electrical stimulation to improve postural balance and plantar sensation among patients with diabetic peripheral neuropathy: a randomized double blinded study. J Diabetes Sci Technol.

[21] Valle G (2021). A psychometric platform to collect somatosensory sensations for neuroprosthetic use. Front Med Technol.

[22] Wannawas N, Subramanian M, Faisal AA. Neuromechanics-based deep reinforcement learning of neurostimulation control in FES cycling. 2021 10th International IEEE/EMBS Conference on Neural Engineering (NER). 2021, pp. 381–384. 10.1109/NER49283.2021.9441354.

[23] Febbo DD et al., Does reinforcement learning outperform PID in the control of FES-induced elbow flex-extension? In 2018 IEEE International Symposium on Medical Measurements and Applications (MeMeA). 2018, pp. 1–6. 10.1109/MeMeA.2018.8438800.

[24] Picillo M, Lozano AM, Kou N, Puppi Munhoz R, Fasano A (2016). Programming deep brain stimulation for Parkinson’s disease: the Toronto western hospital algorithms. Brain Stimul.

[25] Dunkelberger N, Schearer EM, O’Malley MK (2020). A review of methods for achieving upper limb movement following spinal cord injury through hybrid muscle stimulation and robotic assistance. Exp Neurol.

[26] Graczyk EL, Delhaye BP, Schiefer MA, Bensmaia SJ, Tyler DJ (2018). Sensory adaptation to electrical stimulation of the somatosensory nerves. J Neural Eng.

[27] Picillo M, Lozano AM, Kou N, Munhoz RP, Fasano A (2016). Programming deep brain stimulation for tremor and dystonia: the Toronto western hospital algorithms. Brain Stimul.

[28] Louie KH (2021). Semi-automated approaches to optimize deep brain stimulation parameters in Parkinson’s disease. J Neuroeng Rehabil.

[29] Feng X, Greenwald B, Rabitz H, Shea-Brown E, Kosut R (2007). Toward closed-loop optimization of deep brain stimulation for Parkinson’s disease: concepts and lessons from a computational model. J Neural Eng.

[30] Lorenz R (2019). Efficiently searching through large tACS parameter spaces using closed-loop Bayesian optimization. Brain Stimul.

[31] Laferrière S, Bonizzato M, Côté SL, Dancause N, Lajoie G (2020). Hierarchical Bayesian optimization of spatiotemporal neurostimulations for targeted motor outputs. IEEE Trans Neural Syst Rehabil Eng.

[32] Sutton RS, Barto AG (1999). Reinforcement learning. J Cogn Neurosci.

[33] Nagaraj V, Lamperski A, Netoff TI (2017). Seizure control in a computational model using a reinforcement learning stimulation paradigm. Int J Neur Syst.

[34] Basla C, Chee L, Valle G, Raspopovic S (2022). A non-invasive wearable sensory leg neuroprosthesis: mechanical, electrical and functional validation. J Neural Eng.

[35] Petrini FM (2019). Six-month assessment of a hand prosthesis with intraneural tactile feedback. Ann Neurol.

[36] Rognini G (2019). Multisensory bionic limb to achieve prosthesis embodiment and reduce distorted phantom limb perceptions. J Neurol Neurosurg Psychiatry.

[37] Kluger DT (2019). Virtual reality provides an effective platform for functional evaluations of closed-loop neuromyoelectric control. IEEE Trans Neural Syst Rehabil Eng.

[38] Preatoni G, Bracher NM, Raspopovic S. Towards a future VR-TENS multimodal platform to treat neuropathic pain. In 2021 10th International IEEE/EMBS Conference on Neural Engineering (NER), 2021, pp. 1105–1108. 10.1109/NER49283.2021.9441283.

[39] Pozeg P (2017). Virtual reality improves embodiment and neuropathic pain caused by spinal cord injury. Neurology.

[40] Holly R. HTC Vive Flow wants to be your portable VR escape glasses. CNET, 2021. https://www.cnet.com/tech/computing/htc-vive-flow-wants-to-be-your-portable-vr-escape-glasses/. Accessed 01 Aug 2022.

[41] Esposito F. Apple’s AR glasses reportedly coming late 2024 - 9to5Mac. 2022. https://9to5mac.com/2022/06/12/apples-ar-glasses-coming-late-2024/. Accessed01 Aug 2022.

[42] Sharif M, Erdogmus D, Amato C, Padir T. Towards end-to-end control of a robot prosthetic hand via reinforcement learning. In 2020 8th IEEE RAS/EMBS International Conference for Biomedical Robotics and Biomechatronics (BioRob), 2020, pp. 641–647. 10.1109/BioRob49111.2020.9224380.

[43] Mnih V (2015). Human-level control through deep reinforcement learning. Nature.

[44] Mogyoros I, Kiernan MC, Burke D (1996). Strength-duration properties of human peripheral nerve. Brain.

[45] Geddes LA (2004). Accuracy limitations of chronaxie values. IEEE Trans Biomed Eng.

[46] George JA (2019). Biomimetic sensory feedback through peripheral nerve stimulation improves dexterous use of a bionic hand. Sci Robot.

[47] Chai G, Wang H, Li G, Sheng X, Zhu X (2022). Electrotactile feedback improves grip force control and enables object stiffness recognition while using a myoelectric hand. IEEE Trans Neural Syst Rehabil Eng.

[48] Bensmaia SJ, Tyler DJ, Micera S (2020). Restoration of sensory information via bionic hands. Nat Biomed..

[49] Farina D (2021). Toward higher-performance bionic limbs for wider clinical use. Nat Biomed Eng.

[50] Zhang GY (2020). Diabetic peripheral neuropathy increases electrical stimulation threshold of sciatic nerve: a prospective parallel cohort study. Diabetes Metab Syndr Obes.

[51] Keyl C, Held T, Albiez G, Schmack A, Wiesenack C (2013). Increased electrical nerve stimulation threshold of the sciatic nerve in patients with diabetic foot gangrene: a prospective parallel cohort study. Eur J Anaesthesiol.

[52] Cogan SF (2008). Neural stimulation and recording electrodes. Annu Rev Biomed Eng.

[53] Shepard RK, Ellenbogen KA (2009). Leads and longevity: how long will your pacemaker last?. Europace.

[54] Ortiz-Catalan M, Mastinu E, Sassu P, Aszmann O, Brånemark R (2020). Self-contained neuromusculoskeletal arm prostheses. N Engl J Med.

[55] Valle G (2018). Biomimetic intraneural sensory feedback enhances sensation naturalness, tactile sensitivity, and manual dexterity in a bidirectional prosthesis. Neuron.

[56] Charkhkar H, Shell CE, Marasco PD, Pinault GJ, Tyler DJ, Triolo RJ (2018). High-density peripheral nerve cuffs restore natural sensation to individuals with lower-limb amputations. J Neural Eng.

[57] Chandrasekaran S (2020). Sensory restoration by epidural stimulation of the lateral spinal cord in upper-limb amputees. Elife.

[58] Armenta Salas M (2018). Proprioceptive and cutaneous sensations in humans elicited by intracortical microstimulation. Elife.

[59] Raspopovic S (2021). Neurorobotics for neurorehabilitation. Science.

[60] Crema A, Malešević N, Furfaro I, Raschellà F, Pedrocchi A, Micera S (2018). A wearable multi-site system for NMES-based hand function restoration. IEEE Trans Neural Syst Rehabil Eng.

[61] Waschneck B (2018). Optimization of global production scheduling with deep reinforcement learning. Procedia Cirp.

[62] Hester T (2018). Deep q-learning from demonstrations. Proc AAAI Confer Artif Intell..

[63] Lillicrap TP, et al. Continuous control with deep reinforcement learning. *arXiv preprint *arXiv:1509.02971. 2015.

[64] Sharif M et al. Towards End-to-End control of a robot prosthetic hand via reinforcement learning. 2020 8th IEEE RAS/EMBS International Conference for Biomedical Robotics and Biomechatronics (BioRob). IEEE, 2020.

[65] MathWorks Inc. , Deep Q-Network (DQN) agents, https://www.mathworks.com/help/reinforcement-learning/ug/dqn-agents.html.

[66] Raspopovic S (2014). Restoring natural sensory feedback in real-time bidirectional hand prostheses. Sci Transl Med.

