# Supplementary material for: Automated calibration of somatosensory stimulation using reinforcement learning
Source: J Neuroeng Rehabil. 2023 Sep 26;20:131. doi: 10.1186/s12984-023-01246-0 (PMC10523674; doi:10.1186/s12984-023-01246-0)
Supplement: Supplementary file 1 — Additional file 1: Fig S1. RL environment algorithms and offline testing results. Fig. S2. Step-wise evolution of offline simulated RL. Fig S3. Low- and high-level agents states and rewards. Fig S4. Online comparison of RL, BFA, expert and naïve mapping performances. Fig S5. Deep Q-learning training algorithm. Fig S6. Naïve mapping algorithm. Fig S7. Brute force mapping algorithm. [file 12984_2023_1246_MOESM1_ESM.docx]

**Supplementary Information**

**Automated calibration of somatosensory stimulation using reinforcement learning**

**List of Supplementary Materials:**

Fig S1. RL environment algorithms and offline testing results.

Fig. S2. Step-wise evolution of offline simulated RL.

Fig S3. Low- and high-level agents states and rewards.

Fig S4. Online comparison of RL, BFA, expert and naïve mapping performances.

Fig S5. Deep Q-learning training algorithm

Fig S6. Naïve mapping algorithm.

Fig S7. Brute force mapping algorithm

Movie S8: Explanation of the AI-VR calibration platform for sensory feedback

1. **RL Environment**
2. **1.1 Features extraction process**

For each subject and mapping carried out (Tab. S1), 7 features related to the perceived intensity, type and location of the sensation has been collected. The enumeration is given in brackets in the following lines. Starting from the pulse amplitude (PA) (1) and pulse width (PW) (2) values found for the low and high level, the corresponding charge released was calculated as Q = PA * PW (3). Given a plausibly linear relationship between the released charge and the intensity of the sensation evoked by the stimulation, as reported by D’Anna et al. [1], a linear interpolation was performed between these two points in the slope-intercept form (y = mx + q). Since with the same stimulation parameters the sensation evoked on the subjects can be different, this will be reflected in a variation of the coefficients (i.e., *m* (4) and *q* (5)) which define the interpolation line between the two perceived intensity levels. In addition, subject gender (6) and the targeted nerve (7) were also known and considered as features. The corresponding output of these input features was also known from the characterization form fulfilled by the subjects at the end of the mapping and describing the evoked sensation. Four possible levels have been used to label the reported perceived intensity: 1) *Not perceived level* (representing a perceived intensity lower than 2/10); *Low level* (the lowest stimulus charge at which the subject reliably feels a sensation rated as a perceived intensity equal to 2/10); 3) *High level* (stimulus charge at which the sensation becomes close to uncomfortable or painful, rated as a perceived intensity equal to 8/10); *Too high level* (level of perceived intensity higher than 8/10, i.e., painful sensation). The output related to the type of sensation reported was categorized into one of two possible classes namely *comfortable* (i.e., pressure, touch, tingling, pulsation, vibration) and *uncomfortable* (i.e., electricity, warm, cold, pain, twitch). Since the goal of the characterization procedure is to find an electrode position and parameter configuration that elicits a somatotopic sensation minimizing the *in loco* sensation (i.e., under the electrodes) their combination has been considered to define the reported location output. It has been categorized into one of two possible classes namely *somatotopic* and *not somatotopic*. A threshold to establish if the evoked sensation was somatotopic and another to determine if the intensity of the sensation under the electrodes was too high have been defined. The reported output belonged to a somatotopic location if the sensation evoked was simultaneously somatotopic and with an intensity under the electrodes below 5 on a scale of 1 to 10. If either of these two conditions was not met, the output was classified as not somatotopic.

**1.2 Algorithms’ training**

In order to predict the sensation's output in terms of perceived intensity, type and location, three separate machine learning-based models were adopted. The training and validation of such algorithms was performed using the Matlab toolbox Classification Learner for the type output and Regression learner for the perceived intensity and location outputs. A features selection phase was empirically carried out, with the aim of discriminating which subset of predictors led to the maximal validation accuracy on trained models. Once the predictors were identified, they were then used as input features for each model. The toolboxes also allowed the simultaneous testing of different machine learning algorithms. Their output consists in a pre-trained model, of which performances can be assessed performing a 5-fold cross - validation.

Perceived intensity: After empirically testing all the possible combinations of the input features, the best result was achieved considering charge, slope and intercept. These features were then used to predict the perceived intensity level. This result show how the linear relationship existing between charge delivered and perceived sensation intensity [1] is effective in predicting the perceived intensity value ranked from 0 to 10. The machine learning-based algorithm used to predict the four levels of perceived intensity was a linear regression interaction model. At the end of the training the model showed a RMSE equal to exp(-15) in predicting the low- and high- level (Fig. S1B).

Type: The model that showed better results in predicting the two classes that define the type of sensation was a KNN ensemble that achieved an accuracy of 72.3% (Fig. S1B). This result was obtained by considering pulse amplitude, pulse width, slope and intercept as input features (Fig. S1A).

Location: The combinations of the input features through which the best result has been achieved were again pulse amplitude, pulse width, slope and intercept (Fig. S1A). The machine learning-based algorithm that showed the best result with this combination of features was the Gaussian Process Exponential Regression model with an RMSE equal to 0.352. Thereafter, a classification threshold was tuned to create a binary classifier [2-4]. Using a classification threshold equal to 0.3 on the regression result, a classifier with an accuracy of 91.8% was therefore obtained and used to predict the two classes characterizing the location (Fig. S1B).


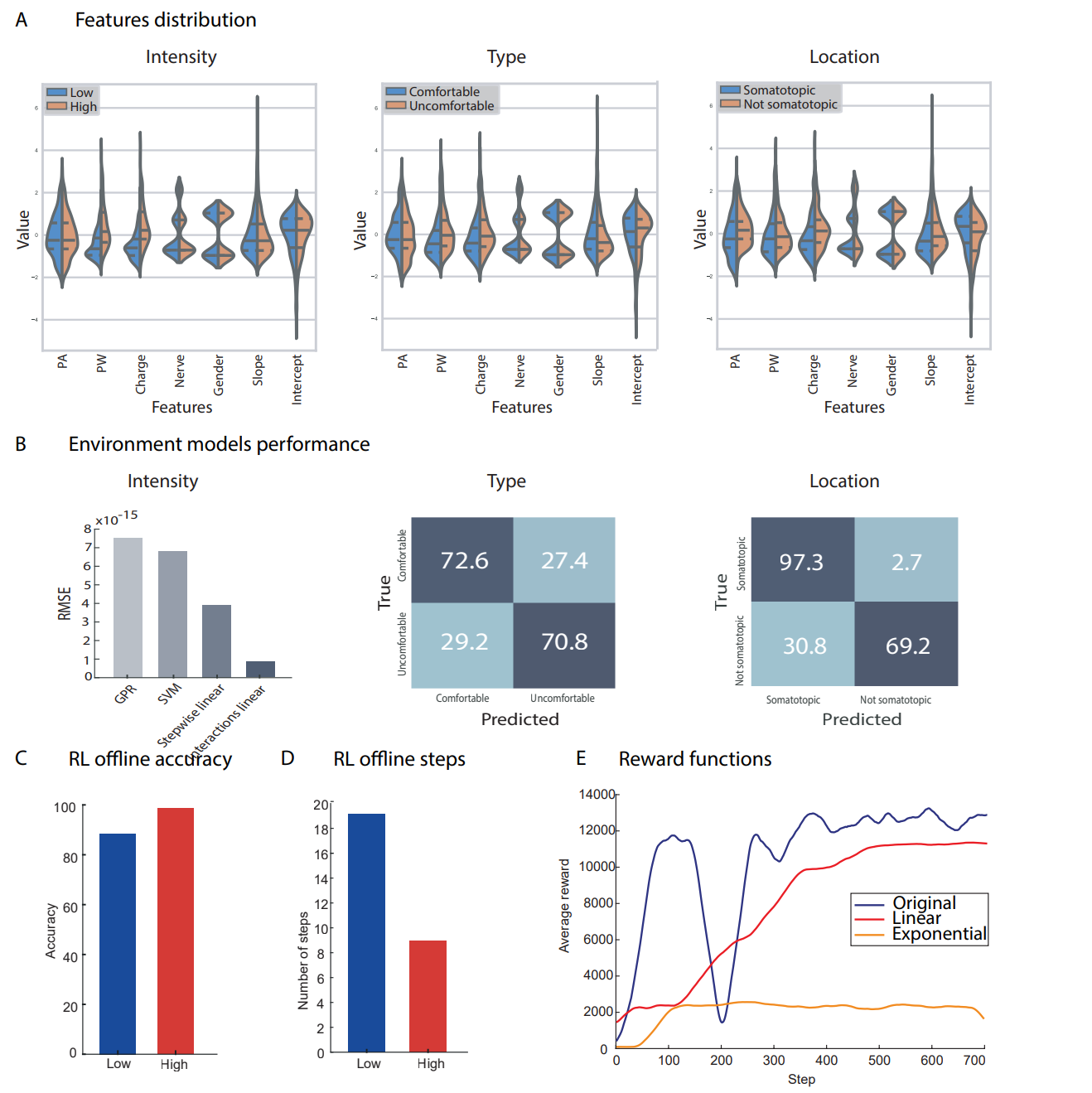


**Fig. S1. RL environment algorithms and offline testing results.** A) Features distribution for the intensity, type and location algorithm selection. B) Performances of the three algorithms characterizing the RL environment. C) Offline accuracy in reaching the target perceived intensity level for the low- and high-level respectively. D) Average number of steps needed to converge during offline simulation for the low- and high-level respectively. E) Performance of three different reward functions tested during the offline stimulation of the RL algorithm (original discrete function, linear ranging from 0 to 12 and exponential function *e^x* with *x* between 0 to 1). The discrete function (Fig S3) shows better performance.


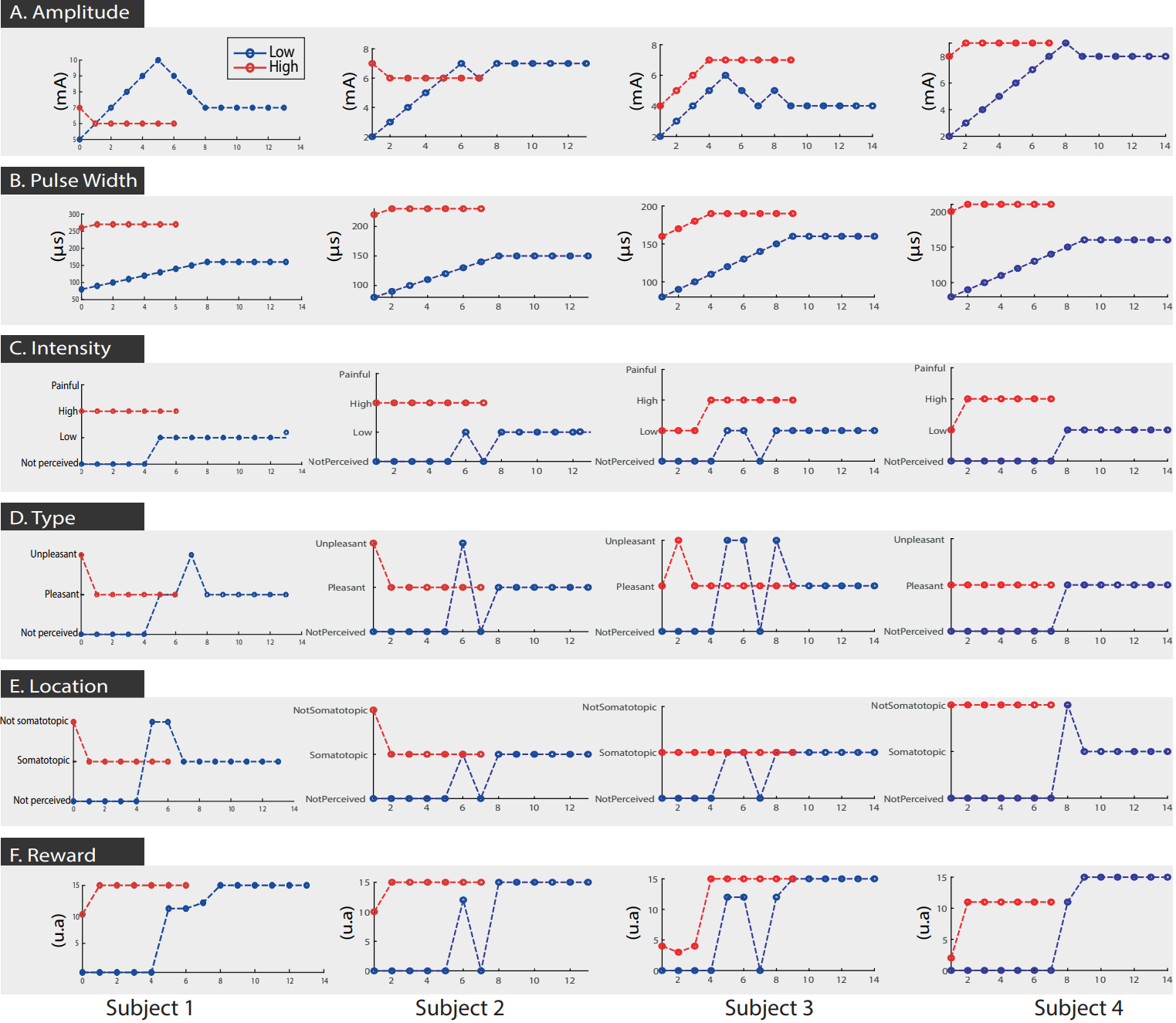


**Fig. S2. Step-wise evolution of offline simulated RL.** Step-wise evolution of amplitude, pulse width, intensity, type, location and reward during the simulation of offline RL on four different subjects. The convergence is set at 5 iterations without changing the actions.

**1.3 States and reward**


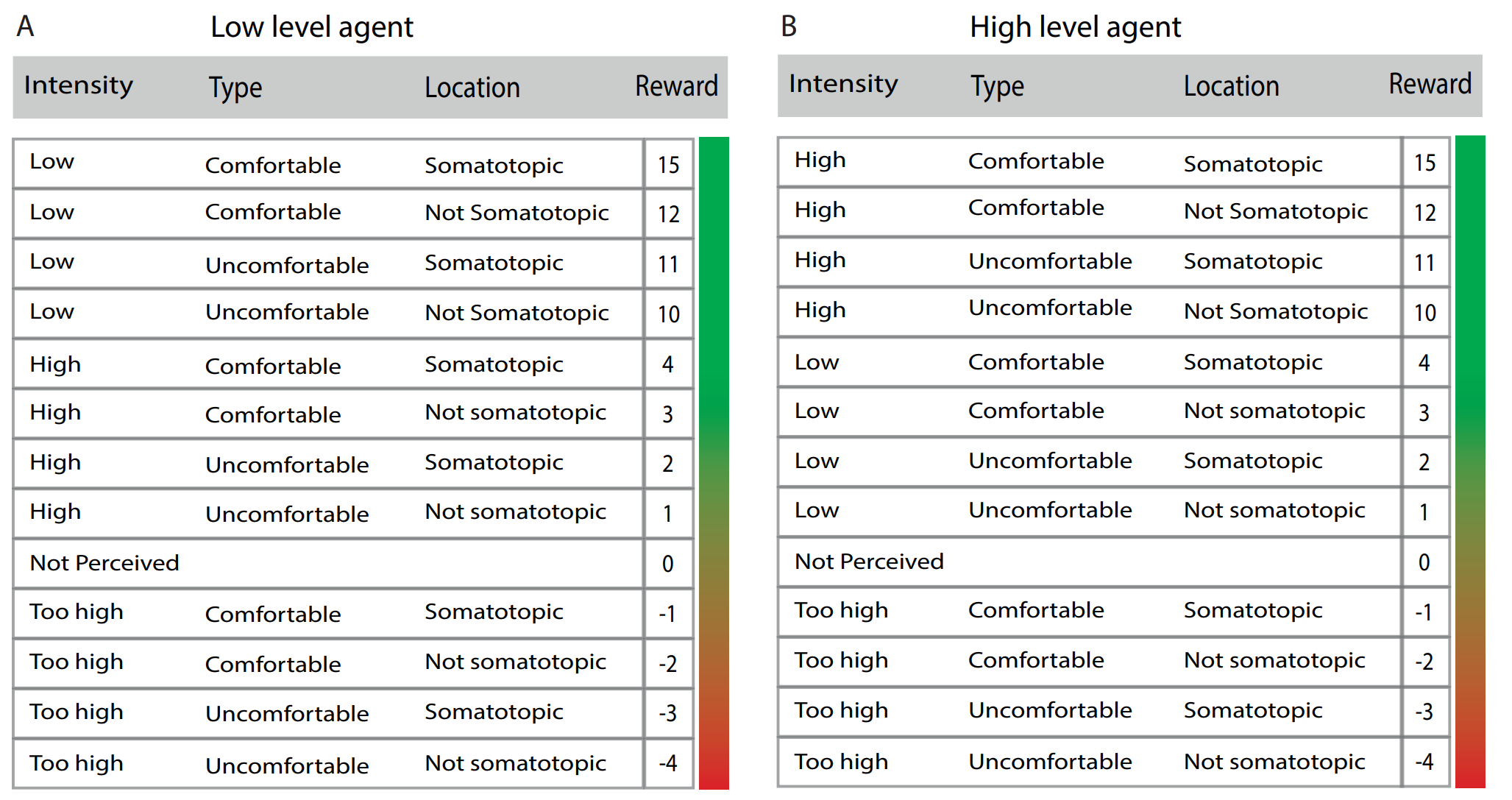


**Fig S3. Low- and high-level agents states and rewards.** A) ranking of the different combination of states (intensity, type and location) and respective rewards for the low-level agent. B) A) ranking of the different combination of states (intensity, type and location) and respective rewards for the high-level agent.

1. **Results healthy subjects’ day 2**


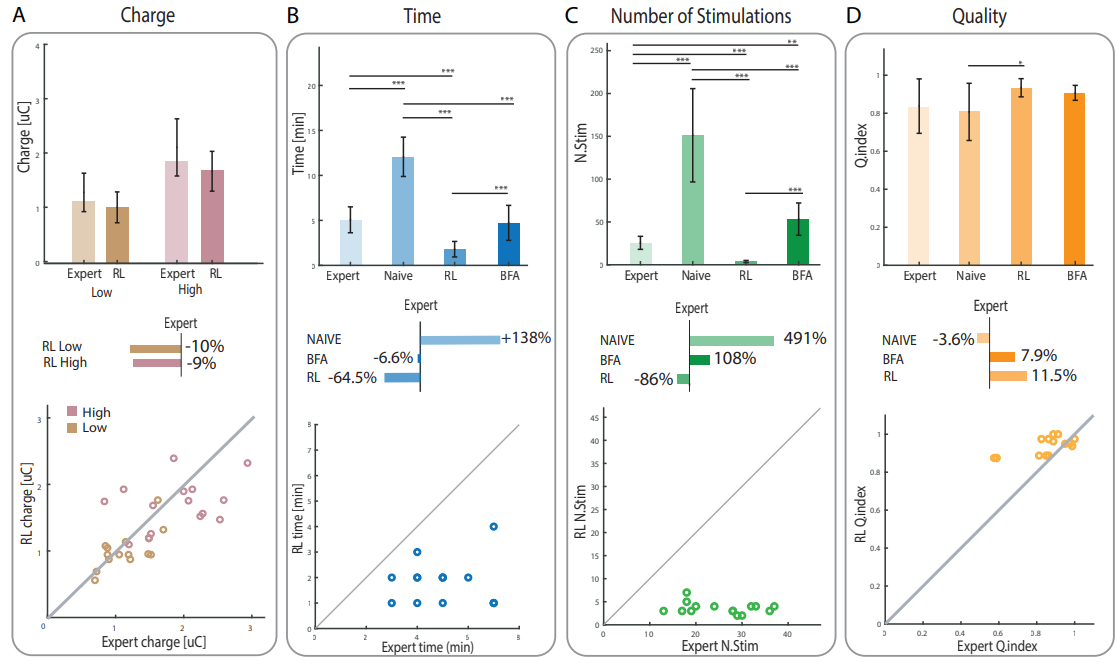


**Fig S4. Online comparison of RL, BFA, expert and naïve mapping performances.** The results during the second day of characterization on healthy subjects are shown. These plots are computed for: (A) final charge released by the stimulation parameters found (divided by low- and high-level calibration), (B) time needed to perform the characterization of the nerve, (C) number of stimulations delivered, and (D) overall sensation quality of the mapping. The bar plots represent the mean values and standard deviation of the measurements of 15 nerves of five independent subjects in the four conditions (p < 0.0083 (*), p < 0.0017 (**), p < 0.00017 (***)). The four conditions were then compared to the expert and expressed as a percentage of the expert performance. The scatter plots represent a direct comparison between the RL and the expert for each trial.

1. **Algorithms and experimental protocol**


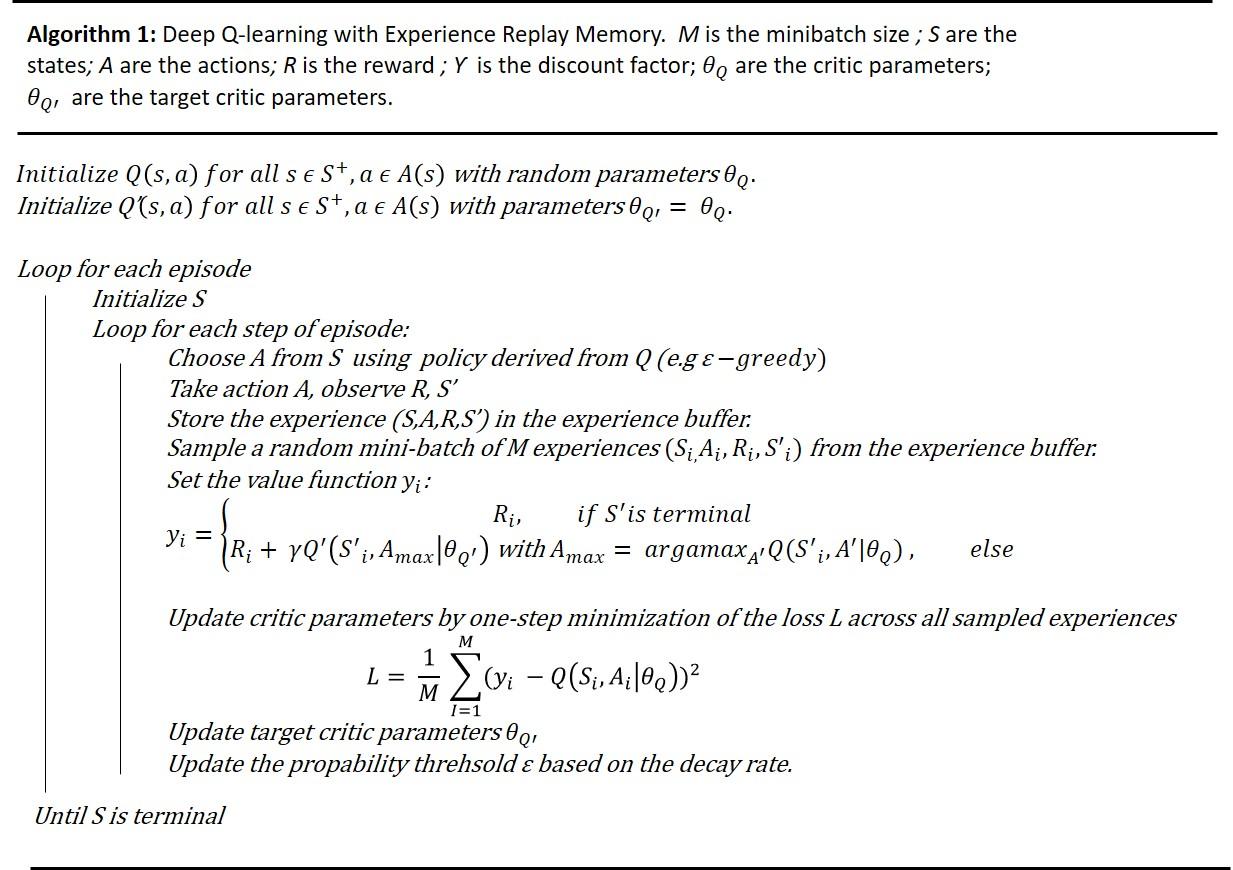


**Fig S5. Deep Q-learning training algorithm.** The RL training algorithm used to train the two DQN agents is represented. The algorithm is a trade-off between exploration and exploitation which led the agents to find optimal solutions quickly. The action’s choice (A) is indeed guided by a probability function (i.e., policy) which sample the states space (S) in order to discover the optimal strategy to reach the target state. The state transition (S 🡪 S’) following the action’s choice is then rated by means of a reward (R) in order to provide the agent with a qualitative information of the choice made. The tuple (S, A, R, S’) therefore represents the RL agent’s experience and the trial-and-error process behind the reinforcement learning training algorithm.


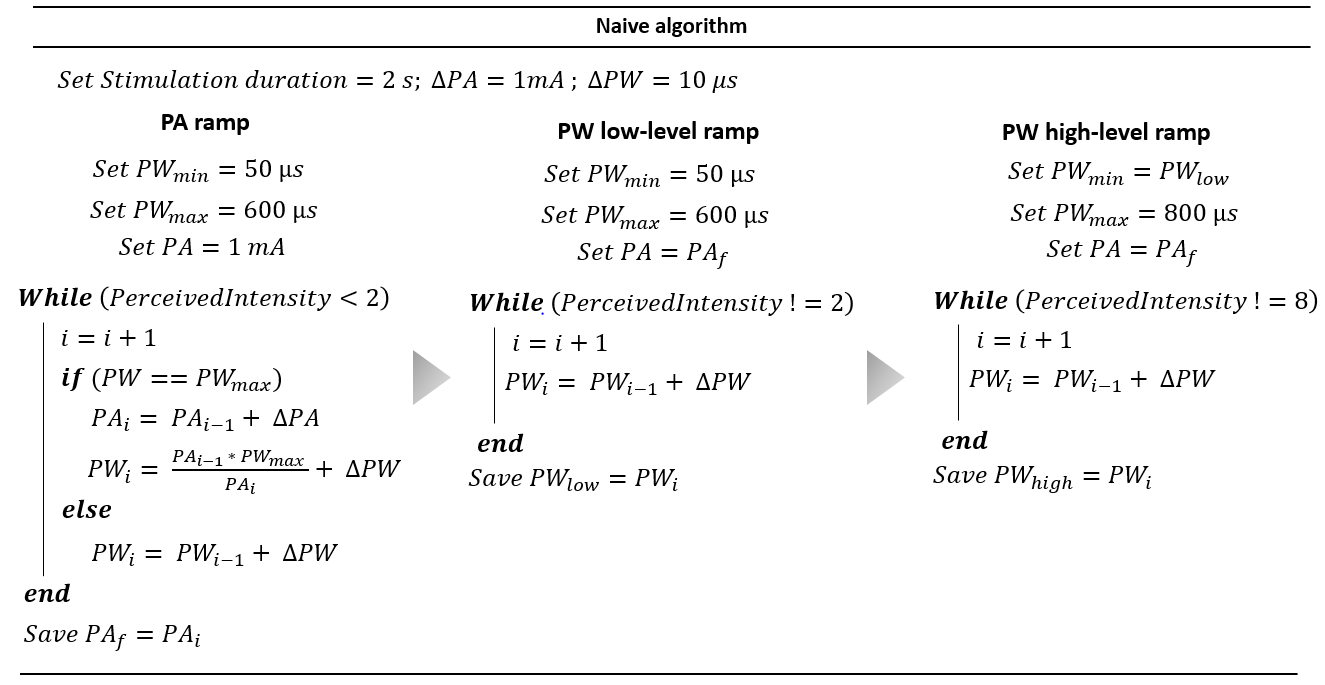


**Fig S6. Naïve mapping algorithm.** The figure shows the protocol used by the naive experimenter to perform the characterization. The first step involved identifying the pulse amplitude value. PW ramps were then performed for a given PA value. Starting from PW = PW_min_, when the max PW value was reached, the PA value was increased and the corresponding starting PW value was calculated. The process was repeated until the subject reported an intensity of the evoked sensation approximately equal to 2 on a scale of 0 out of 10. The final PA value was then saved and used for the subsequent steps. The second step involved identifying the PW value for the low-level. Keeping the PA value found in step 1 fixed, PW ramps were performed until the subject reported an intensity of the evoked sensation equal to 2 on a scale of 0 out of 10. The PW value corresponding to the low-level was then saved. This value was then used in step 3 as the starting point of the PW ramps in order to identify the high-level PW value. The subject was asked to report when the intensity of the evoked sensation was equal to 8 on a scale of 0 out of 10. At that point the corresponding PW value was saved, and the mapping was finished.


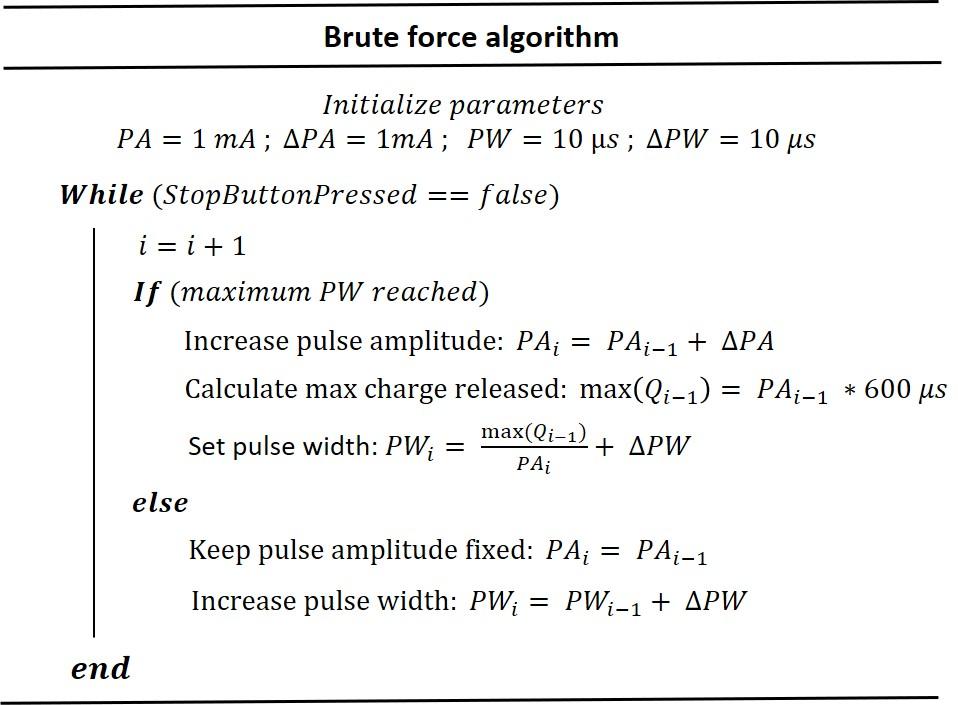


**Fig S7. Brute force mapping algorithm.** The paradigm behind the implementation of the brute force algorithm (BFA) is represented. Two arrays containing pulse amplitude and pulse width values ​​ranging respectively from 1mA to 16mA (ΔPA = 1 mA) and from 10 µS to 600 µS (ΔPW = 10 µS) have been defined. The subject, immersed in the same virtual environment used for the implementation of the RL algorithm, began the characterization using the appropriate start button. The parameters were initialized starting from the lowest value found to elicit a low-level sensation in the offline dataset (560 nC, PA=1mA; PW=560 µs). The second day, the BFA was initialized selecting the PA found in day 1 and starting from the lowest PW, to ensure that the low-level sensation was achieved despite possible decreasing of threshold. Then, the algorithm started to search for the optimal stimulation parameters by linearly increasing the pulse width value while keeping the pulse amplitude value fixed. If the upper limit of the PW array was reached, the maximum charge released was calculated, the pulse amplitude value was increased and the PW value was defined accordingly. The subject was then asked to stop the stimulation when the intensity of the evoked sensation was equal to 2 on a scale of 1 to 10. He then described the type of sensation evoked, the location and intensity of the sensation under the electrodes as well as took place in the AI-based mapping platform. Starting from the parameters found for the low-level, the same process was repeated to find the high-level parameters.

**Expert mapping algorithm**

Unlike the Naive and BFA condition, the expert condition does not follow a defined protocol in performing the characterization. Indeed, the expert adjusts the stimulation parameters based of his/her own experience. Similarly, to the Naive conditions, he / she begins the characterization by performing a pulse amplitude ramp at a fixed PW value but with personalized choice of starting PA and PW step. Once the PA value that elicit a somatotopic and minimized in loco sensation has been identified, the pulse width modulation is carried out so that two levels of perceived intensity are identified (i.e., low and high level). The subject is therefore asked to report when the intensity of the perceived sensation is equal to 2 on a scale from 0 to 10 and the corresponding PW value is saved. The process is then repeated to identify the PA and PW values which elicit a sensation of intensity equal to 8 on the same scale. The initial and final values ​​of the ramps and the steps are freely chosen by the expert.

**Movie S1: Explanation of the AI-VR calibration platform for sensory feedback**

The subject starts the calibration using automatic initialization from the RL algorithm. Then, the subject places the electrodes superficially in correspondence of each specific nerve and enters the VR where is immersed in a user-friendly virtual scenario. The stimulator stimulates each of 3 channels with the received parameters. When the stimulation ends, the subject describes the perceived sensory feedback through questionnaires made in the VR scenario, which include: 1) the perceived intensity, 2) type, 3) intensity perceived under the electrodes and 4) location of sensation on the foot. The answers are sent back to the RL algorithm which updates the neurostimulation parameters accordingly.

[1] E. D’Anna *et al.*, “A somatotopic bidirectional hand prosthesis with transcutaneous electrical nerve stimulation based sensory feedback,” *Sci Rep*, vol. 7, no. 1, p. 10930, Sep. 2017, doi: 10.1038/s41598-017-11306-w.

[2] Kirasich, Kaitlin, Trace Smith, and Bivin Sadler. "Random forest vs logistic regression: binary classification for heterogeneous datasets." *SMU Data Science Review* 1.3 (2018): 9.

[3] Dreiseitl, Stephan, and Lucila Ohno-Machado. "Logistic regression and artificial neural network classification models: a methodology review." *Journal of biomedical informatics* 35.5-6 (2002): 352-359.

[4] Maalouf, Maher. "Logistic regression in data analysis: an overview." *International Journal of Data Analysis Techniques and Strategies* 3.3 (2011): 281-299.
